# The Interaction of Selenium with Chemotherapy and Radiation on Normal and Malignant Human Mononuclear Blood Cells

**DOI:** 10.3390/ijms19103167

**Published:** 2018-10-15

**Authors:** Richard J. Lobb, Gregory M. Jacobson, Ray T. Cursons, Michael B. Jameson

**Affiliations:** 1 Tumour Microenvironment Laboratory, QIMR Berghofer Medical Research Institute, Herston, QLD 4006, Australia; Richard.Lobb@qimrberghofer.edu.au; 2 Department of Biological Sciences, University of Waikato, Hamilton 3216, New Zealand; greg.jacobson@waikato.ac.nz (G.M.J.); ray.cursons@waikato.ac.nz (R.T.C.); 3 Oncology Department, Waikato Hospital, Hamilton 3204, New Zealand; 4 Waikato Clinical Campus, Faculty of Medical and Health Sciences, University of Auckland, Hamilton 3204, New Zealand

**Keywords:** selenium, glutathione, malignant, viability, DNA damage, ER stress

## Abstract

Selenium, a trace element with anticancer properties, can reduce harmful toxicities of chemotherapy and radiotherapy without compromising efficacy. However, the dose-response relationship in normal versus malignant human cells is unclear. We evaluated how methylseleninic acid (MSA) modulates the toxicity and efficacy of chemotherapy and radiation on malignant and non-malignant human mononuclear blood cells in vitro. We specifically investigated its effects on endoplasmic reticulum stress induction, intracellular glutathione concentration, DNA damage and viability of peripheral blood mononuclear cells and THP1 monocytic leukaemia cells in response to radiation, cytosine arabinoside or doxorubicin chemotherapy. MSA, at lower concentrations, induced protective responses in normal cells but cytotoxic effects in malignant cells, alone and in conjunction with chemotherapy or radiation. However, in normal cells higher concentrations of MSA were directly toxic and increased the cytotoxicity of radiation but not chemotherapy. In malignant cells higher MSA concentrations were generally more effective in combination with cancer treatments. Thus, optimal MSA concentrations differed between normal and malignant cells and treatments. This work supports clinical reports that selenium can significantly reduce dose-limiting toxicities of anticancer therapies and potentially improve efficacy of anticancer treatments. The optimal selenium compound and dose is not yet determined.

## 1. Introduction

Selenium (Se) is an essential trace element that is extensively studied in the prevention of numerous malignancies [1], although the majority of research on Se has focused on providing adequate nutritional intake in populations that have inherently low Se intake [2]. However, substantial preclinical data suggests that Se compounds, in supranutritional doses, have direct anticancer effects, mediated by various mechanisms including oxidative capability and modulation of immunological responses, angiogenesis, protein confirmation and DNA repair pathways [3,4]. These same mechanisms allow selenium compounds to act in synergy with cancer therapies and increase the efficacy of these treatments while reducing their normal tissue toxicities, as reviewed by Evans et al. [4]. Se compounds, when added to chemotherapy, resulted in improved tumour response rates and cures in human tumour xenograft animal models and reduced organ-specific toxicity [5,6,7]. Some aspects of these findings have been replicated in clinical trials, with various Se compounds ameliorating the toxicity of chemotherapy or radiotherapy [8,9,10,11,12,13,14,15,16,17], although the trials were not powered to evaluate overall treatment efficacy. These promising results argue for the initiation of larger clinical trials that can definitively assess the contributions of Se compounds to modulating both efficacy and toxicity of chemotherapy and radiation [4,18].

There is unquestionably a major unmet need in this regard. Despite many advances in supportive care, the toxicities of chemotherapy and radiotherapy still limit their efficacy, utility and acceptability to patients and clinicians, and result in poor quality of life for patients, treatment-related deaths and inadequate outcomes [19]. Apart from antiemetics and haemopoietic growth factors, few agents substantially prevent these toxicities, many are poorly-tolerated, and some reduce toxicities while compromising anticancer efficacy [20,21,22]. In contrast, Se compounds offer the potential, at optimum doses, of being well-tolerated agents that can improve both cancer outcomes and treatment toxicities.

In one study, Se-methyl-selenocysteine was more effective and dose-potent than seleno-l- methionine or sodium selenite in reducing cytotoxic chemotherapy-related mortality and augmenting its anticancer activity [6]. This may relate to the in vivo ability of Se-methyl-selenocysteine to directly generate methylselenol, a compound that is considered the active moiety for the observed effects of Se compounds in cancer cells [23,24,25,26,27]. In preclinical models Se-methyl-selenocysteine dosed at 0.2 mg/mouse/day optimises the mechanisms that mediate protection of normal tissues while enhancing tumour cytotoxicity [5,6,28]. In humans, however, this dose-response relationship has not been well-characterised, and thus the optimal type and dose of Se for use in clinical trials has not yet been determined [4]. Therefore, there is a need to provide a framework for characterising the divergent biological effects of Se in normal and malignant cells in humans, to inform future trials evaluating Se compounds in conjunction with anticancer treatments.

This investigation was undertaken to evaluate whether peripheral blood mononuclear cells (PBMCs) from healthy blood donors and a comparable malignant human cell line, THP1 monocytic leukaemia, could serve as an in vitro model to investigate the differential effects of Se on normal and malignant human mononuclear cells. Se has been previously shown to enhance apoptosis through the induction of endoplasmic reticulum (ER) stress in cancer cells [29], therefore we evaluated the induction of ER stress in both normal and malignant cells in response to Se treatment. Given that ER stress signalling can be induced in response to oxidative triggers, we also investigated the impact of Se on intracellular glutathione levels [30,31,32], a key component in maintaining redox homeostasis in the cell, and how this influences DNA damage and viability of normal and malignant cells to cytotoxic chemotherapy or radiation [33,34,35].

Instead of Se-methyl-selenocysteine, which does not generate methylselenol in vitro*,* we used methylseleninic acid (MSA), which directly provides methylselenol through non-enzymatic reduction, and enabled us to directly evaluate the impact of this active metabolite of Se compounds [25,27]. We used MSA at Se concentrations (2.5, 5 and 15 µM) that could be achieved in plasma in subsequent clinical trials, and were comparable to plasma levels in mice at effective doses [6]. MSA was used alone or in combination with cytotoxic chemotherapy drugs or gamma radiation to evaluate their interactions in normal and malignant cells.

We demonstrate that Se has divergent effects in normal and malignant human mononuclear cells, protecting normal cells from chemotherapy and radiation toxicity while enhancing their therapeutic effects against malignant cells. In this model we were also able to use analytical methods to demonstrate changes in biological pathways that mediate these effects of Se compounds, which could be incorporated into future clinical trials.

## 2. Results

### 2.1. Methylseleninic Acid (MSA) Induces Endoplasmic Reticulum (ER) Stress in Normal and Malignant Cells But Differentially Modulates Apoptosis

**Figure 1:**
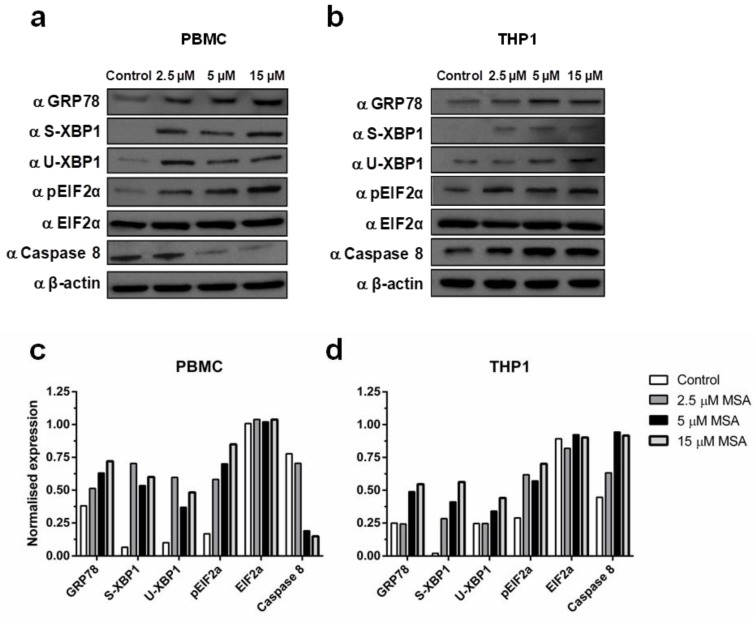
Selenium induces endoplasmic reticulum (ER) stress response in normal and malignant cells. (**a**) Concentration-dependent increase in ER stress proteins and decrease in caspase-8 in peripheral blood mononuclear cells (PBMCs) with 2.5, 5 and 15 µM methylseleninic acid (MSA) at 6 h; (**b**) Concentration-dependent increase in both ER stress proteins and caspase-8 in THP1 cells; (**c**,**d**) Quantification of protein expression in PBMC and THP1 cells.

To investigate the induction of ER stress in normal and malignant cells we measured the cellular expression of 78 kDa glucose-regulated protein (GRP78) and phosphorylated eukaryotic initiation factor 2-alpha (phospho-EIF2α), and splicing of X-box binding protein 1 (XBP1), in response to exposure to increasing concentrations of MSA for 6 h. MSA induced ER stress in both normal and malignant cells, which was seen through an increase in the expression of GRP78, as well as an increase in the splicing of XBP1 (spliced: S-XBP1; unspliced: U-XBP1) and phosphorylation of EIF2α (Figure 1). Interestingly, when we assessed the effect of MSA on the apoptotic response induced by ER stress we found different patterns between normal and cancer cells (Figure 1). Caspase-8 was down-regulated by MSA in a concentration-dependent manner in normal PBMCs yet was upregulated in malignant THP1 cells at the same concentrations, with the maximal differential impact between normal and malignant cells at 5 µM MSA (Figure 1).

### 2.2. MSA Has a Divergent Impact on Glutathione (GSH) Levels in Normal and Malignant Cells

**Figure 2:**
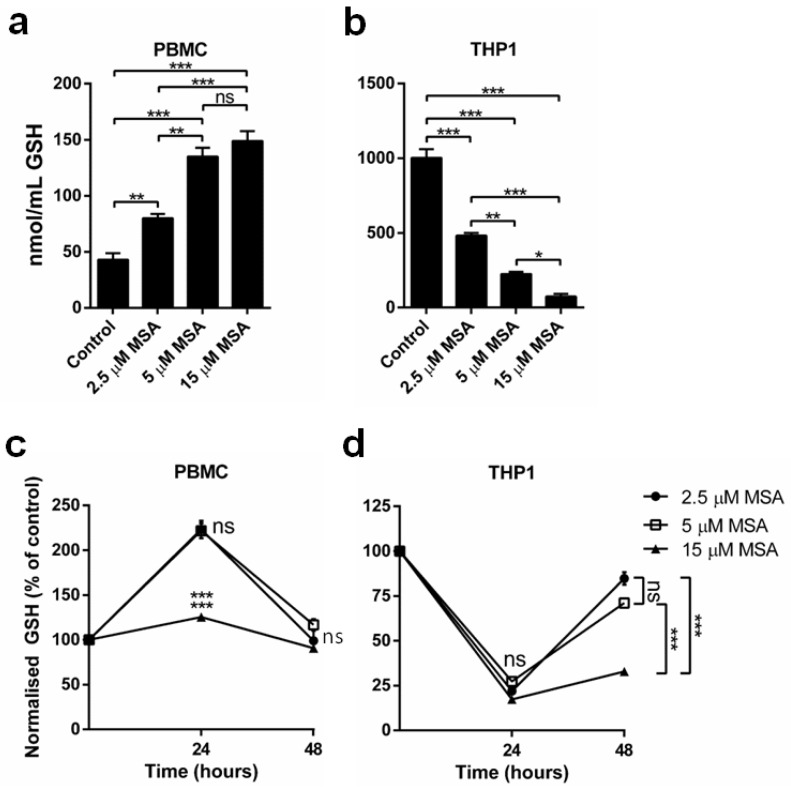
MSA has divergent impact on glutathione (GSH) levels in normal and malignant cells. (**a**,**b**) GSH quantification in PBMC and THP1 cells demonstrates that MSA significantly reduces GSH levels in THP1 cells and significantly increases GSH levels in PBMCs after 6 h (*n* = 5, ± SEM); (**c**,**d**) Timeline of GSH levels in PBMCs and THP1 cells after MSA treatments demonstrates GSH alterations are maintained for up to 24 h. *n* = 3, ± SEM, * *p* < 0.05, ** *p* < 0.01, *** *p* < 0.001, ns, not significant.

To investigate the link between ER stress and generated oxidative stress we measured intracellular total GSH levels in normal and malignant cells. At 6 h we observed differential effects of MSA in normal and malignant cells (Figure 2a). MSA significantly increased total GSH levels in PBMC (Figure 2a) after 6 h in a concentration-dependent manner (a protective response). Conversely, THP1 cells had a baseline GSH level approximately 40-fold higher than PBMCs that was significantly reduced by MSA in a concentration-dependent manner after 6 h (Figure 2b).

We then tested the duration of the MSA-induced alteration on GSH levels in normal and malignant cells. The increase in GSH observed in PBMCs after 6 h of MSA treatment at 2.5 and 5 µM was maintained at 24 h but returned to baseline levels at 48 h (Figure 2c). However, at 15 µM MSA, the GSH concentration was less elevated at 24 h than at 6 h and also returned to baseline levels at 48 h. In THP1 cells, the depletion of GSH at 24 h was still significant but not concentration-dependent, whereas at 48 h the return of GSH levels towards baseline values was greater with 2.5 and 5 µM compared with 15 µM MSA (Figure 2d).

**Figure 3:**
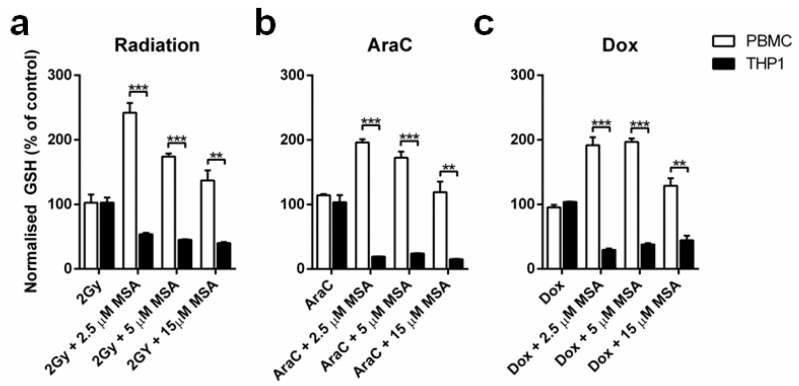
MSA-induced GSH alterations are maintained in the presence of therapeutic treatments. (**a**–**c**) GSH levels are significantly elevated in PBMCs at 24 h after radiation, AraC or Dox treatment, whereas GSH levels are significantly reduced in THP1 cells 24 h after treatment. *n* = 3, ± SEM, ** *p* < 0.01, *** *p* < 0.001.

Next, we investigated if the MSA-induced GSH response in cells was maintained at 24 h after radiation and chemotherapy treatment. The GSH increase in normal PBMCs was maintained at 24 h when cells were also treated with 2 Gy radiation, cytosine arabinoside (AraC) or doxorubicin (Dox), though the maximum benefit was achieved with 2.5 µM MSA (Figure 3a–c). Furthermore, the depletion of GSH by MSA in malignant THP1 cells was still significantly reduced at 24 h after radiation and chemotherapy treatment, again without the advantage of higher MSA concentrations (Figure 3a–c).

### 2.3. MSA Reduces DNA Damage in Normal Cells While Increasing DNA Damage in Malignant Cells

**Figure 4:**
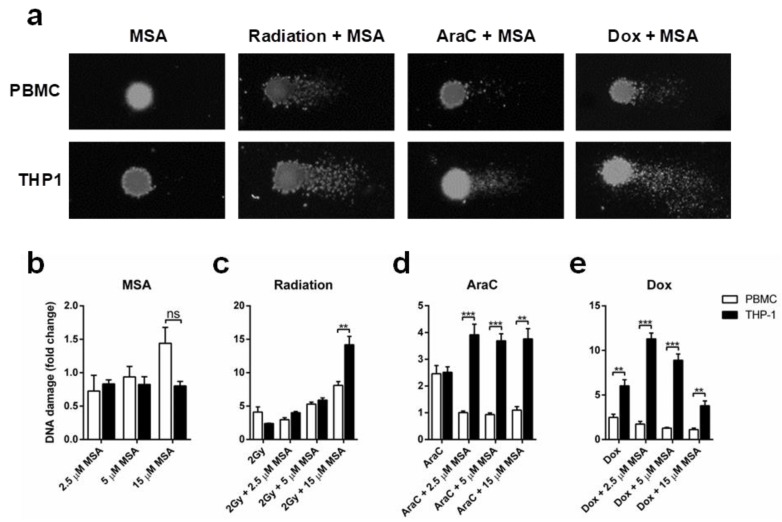
Selenium protects normal cells from DNA damage while enhancing DNA damage in malignant cells. (**a**) Representative image of comet assay (400× magnification) in PBMCs and THP1 cells treated with MSA alone or MSA in combination with radiation, AraC or Dox; (**b**) Quantification of DNA damage in PBMCs and THP1 cells: treatment with MSA 15 µM, but not lower concentrations, slightly increased DNA damage levels in normal but not in malignant cells; (**c**) DNA damage is increased in both PBMCs and THP1 cells exposed to 2 Gy radiation; (**d**,**e**) MSA is significantly protective against DNA damage in PBMCs while significantly increasing DNA damage in THP1 cells treated with AraC or Dox. *n* = 3, ± SEM, ** *p* < 0.01, *** *p* < 0.001, ns, not significant.

Given the divergent effects of MSA on apoptosis induction and GSH expression in normal and malignant cells, we investigated if MSA would protect normal cells from DNA damage due to radiation or chemotherapy, while potentiating the DNA-damaging efficacy of these treatments in malignant cells. Using the comet assay (Figure 4a), this differential effect was pronounced with chemotherapy but not radiation. Treatment with MSA alone at the highest concentration, 15 µM, slightly increased DNA damage levels in normal cells but not in malignant cells, though the lower concentrations had no such effect (Figure 4b).

As expected, when PBMCs and THP1 cells were exposed to 2 Gy radiation DNA damage was elevated compared to controls (Figure 4c). While MSA at 2.5 µM reduced radiation-induced DNA damage in PBMC but not THP1 cells, higher concentrations of MSA progressively increased radiation-induced DNA damage in both normal and malignant cells (Figure 4c). DNA damage was significantly increased in PBMCs and THP1 cells when treated with AraC, however adding MSA protected the normal cells while increasing DNA damage in the malignant cells, without a clear concentration dependency (Figure 4d). Dox-induced DNA damage in PBMCs was not potentiated by MSA, while in contrast MSA increased Dox-induced DNA damage in THP1 cells (Figure 4e). However, this effect on THP1 cells was maximal at 2.5 µM MSA, and diminished at higher concentrations (Figure 4e).

### 2.4. MSA Treatment Protects Normal Cells While Potentiating Cell Death in Malignant Cells

**Figure 5:**
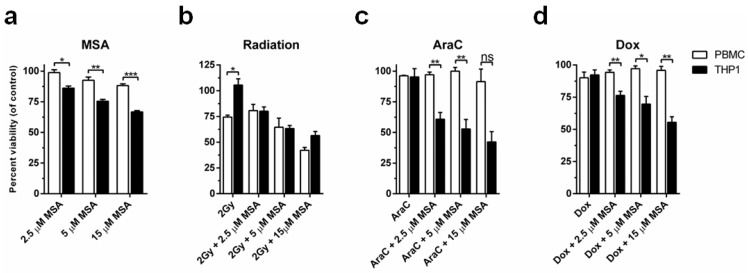
MSA protects normal cells and results in elevated cell killing of malignant cells after 48 h. (**a**) MSA treatment alone significantly reduces cell viability of malignant THP1 cells compared to normal PBMCs; (**b**) MSA does not significantly alter cell viability of PBMCs compared to THP1 cells in response to radiation; (**c**,**d**) MSA significantly protects normal PBMCs from cell death while enhancing the therapeutic activity of AraC or Dox in THP1 cells. *n* = 3 ± SEM, * *p* < 0.05, ** *p* < 0.01, *** *p* < 0.001, ns, not significant.

We next investigated if the differences in DNA damage culminated in differences in cell viability. MSA alone significantly reduced the viability of THP1 cells with increasing MSA concentrations compared to PBMCs (Figure 5a). Although 2 Gy radiation alone did not affect viability of THP1 cells, adding MSA to radiation significantly reduced THP1 cell viability (Figure 5b). In agreement with the DNA damage induced by radiation, the addition of MSA to this treatment further reduced the viability of PBMCs (Figure 5b). However, when we assessed the combination of MSA with AraC or Dox we found significant differences (Figure 5c,d). Treatment with MSA at all concentrations provided significant protection of PBMCs while progressively increasing toxicity in THP1 cells in response to AraC or Dox treatment (Figure 5c,d).

## 3. Discussion

The toxicity of anticancer therapies is a major ongoing clinical issue and developing agents that usefully modulate the toxicity and efficacy of chemotherapy and radiotherapy without compromising their efficacy is important. Preclinical work and some clinical trials suggest that Se compounds can achieve this, though the Se compounds and doses used have varied widely [4,6]. In the present study, we used an in vitro model of normal and malignant human mononuclear blood cells to investigate the dose-response relationship of Se in modulating the efficacy and toxicity of cancer treatments. We have shown important differences between normal and malignant cells in the dose-response relationship of Se to biological mechanisms that mediate cell survival and response to cancer treatments.

Se compounds have previously been shown to induce ER stress in a concentration- and time-dependent manner in prostate cancer cell lines, which leads to apoptosis in malignant cells [29]. In this study, we found that MSA induced apoptosis through caspase-8 expression in THP1 cells while reducing caspase-8 in PBMCs, in agreement with previous studies that have shown Se compounds induce apoptosis through caspase-8 activation [36]. Moreover, caspase-8-mediated apoptosis has been demonstrated to mediate the therapeutic synergy of Se compounds and chemotherapy treatment in various cancer settings [37,38]. The reduction in caspase-8 induced by MSA in PBMC in this study is consistent with the clinical data that Se compounds, at tested doses, are protective of normal tissues [10,16].

ER stress has been demonstrated to induce reactive oxygen species generation [39]. This results in the depletion of intracellular GSH, causing the cellular environment to become more oxidized, which is associated with increased apoptosis and necrosis [40,41,42]. GSH contributes to cellular resistance to anticancer treatments through covalent binding and inactivation of drugs [43,44,45,46,47]. Thus the 40-fold higher initial concentration of GSH present in THP-1 cells compared to PBMCs would protect the malignant cells against cytotoxic therapies, whereas the MSA-induced severe depletion of GSH in malignant cells shown in this study may contribute to the increased sensitivity to these treatments with MSA. These results are consistent with work showing that Se compounds inhibit the cisplatin-induced increase in GSH in ovarian cancer cells, thereby preventing chemoresistance [32]. These malignant cells may also have been sensitised to the effects of MSA due to their high concentrations of GSH, as GSH is a cofactor in the metabolic reduction of MSA to methylselenol [31]. This may not be relevant to Se compounds that generate methylselenol through other mechanisms.

Contrary to the effect of MSA seen in malignant cells, it induced a significant increase in GSH in normal cells, which is expected to protect them against cancer therapies. The simultaneous increase in GSH in normal cells and depletion of GSH in malignant cells may contribute to improving the therapeutic ratio of cancer treatment by reducing normal tissue toxicities while increasing the anticancer efficacy. This effect on GSH may mediate, at least in part, the observed ability of Se compounds to reduce the toxicity of chemotherapy and radiation in normal tissues [10,16].

Previous in vitro and in vivo studies have shown that DNA damage and inducible DNA damage is reduced with Se [33,34]. While we demonstrated that MSA reduced chemotherapy-induced DNA damage in normal cells, it was ineffective in protecting them against radiation-induced DNA damage and cytotoxicity. Of particular concern, at the highest concentration tested (15 µM), MSA significantly increased DNA damage from radiation in PBMC. In contrast, all concentrations of MSA increased the DNA damage and cytotoxicity of radiation and chemotherapy in the malignant cells.

This study supports previous work that demonstrated the potential therapeutic benefit of using Se in conjunction with cancer therapeutics, due to its differential effects on chemotherapy- or radiation-treated normal cells relative to malignant cells [48]. It is encouraging in this study that MSA generally protected normal cells while sensitising malignant cells to cytotoxic therapies, and that it informs about mechanisms that plausibly contribute to the reduction of clinically-significant toxicities seen in clinical trials with Se supplementation during cancer treatments [10,11,12,14].

A very important concern, however, has been raised by this study: in PBMCs the highest concentration of MSA proved toxic, and increased the cytotoxicity and DNA damage from radiation. This could increase the potential for second malignancies and other late complications of radiation, especially if using inorganic forms of Se that are associated with increased genotoxicity compared to several organic forms [49]. These outcomes have not been mentioned in clinical trials to date but the numbers evaluated have been small and follow-up is limited.

There is always a tension in cancer treatment between maximising efficacy while managing toxicities [19]. While Se has considerable and important potential to widen this usually narrow therapeutic window, data from this study strongly suggests that following the traditional cytotoxic therapy paradigm of using the maximum tolerated dose [50,51] may be inappropriate with Se compounds. However, our data also suggests that these interactions are treatment-specific, with greater vulnerability of normal cells when using the highest concentrations of MSA with radiation, but continued protection of normal cells from chemotherapy by MSA at all concentrations. Furthermore, there were marked differences in the concentration-dependence of the improved anticancer effects of chemotherapy or radiation with MSA on malignant THP1 cells. More modest concentrations of MSA proved equally effective to the highest one in terms of inducing ER stress and reducing GSH levels from radiation or chemotherapy, and at inducing DNA damage with chemotherapy drugs. The highest MSA concentration, however, was most effective at inducing DNA damage with radiation and at augmenting the cytotoxicity of radiation or chemotherapy.

## 4. Materials and Methods

### 4.1. Mononuclear Cell Isolation

PBMCs were isolated from buffy coats obtained from blood donations given by healthy individuals, and supplied by the New Zealand Blood Service at Waikato Hospital, Hamilton, New Zealand. Ethical approval for their use was granted by the Northern Y Health and Disability Ethics Committee (reference NTY/10/08/065/AM01, 16 August 2011). The mononuclear cell fraction was isolated via density gradient centrifugation using Histopaque^®^ (St. Louis, MO, USA).

### 4.2. Cell Culture

PBMCs and THP1 cells were cultured in RPMI-1640 medium supplemented with 10% FBS, 1% penicillin (10,000 units/mL) and streptomycin (10,000 µg/mL) at 37 °C in 5% CO_2_. Both cell lines were incubated either in the presence of MSA (2.5, 5 and 15 µM), cytosine arabinoside (AraC; 5 ng/mL), or doxorubicin (Dox; 20 nM) alone, as well as the combination of MSA and AraC or Dox. To assess the response to radiation, cells were irradiated with a total of 2 Gy with or without MSA. Cells were incubated with MSA for 6 h prior to treatment with chemotherapy or radiation.

### 4.3. Western Blot Analysis

Western blotting was carried out as previously described [52]. Briefly, total cell protein was isolated using radioimmunoprecipitation assay (RIPA) buffer (50 mM Tris pH 7.4, 150 mM NaCl, 1% Triton-X-100, 1% Na-deoxycholate, 0.1% sodium dodecyl sulfate (SDS), 1 mM ethylenediaminetetraacetic acid (EDTA), phosphatase inhibitors and protease cocktail inhibitors (Sigma Aldrich, St. Louis, MO, USA), and 1 mM phenylmethanesulfonylfluoride). Proteins were resolved by SDS-polyacriliamide gel (PAGE), transferred to nitrocellulose membranes, blocked in 5% non-fat powdered milk in tris-buffered saline-tween (TBS-T) and probed with antibodies. Protein bands were detected using a FUJIFILM Intelligent dark box II LAS-1000 system.

### 4.4. Measurement of GSH

Glutathione (GSH) assay kit was purchased from Sigma-Aldrich (St. Louis, MO, USA). The assay was carried out according to the manufacturer’s directions. Total GSH was determined using a kinetic assay that measures the reduction of 5,5′-dithiobis-(2-nitrobenzoic) acid (DTNB) to 5-thio-2-nitrobenzoic acid (TNB) at 412 nm.

### 4.5. Comet Assay

DNA damage was assessed with the comet assay as previously described [53]. Preparation of slides was carried out by coating a pre-agarose-coated slide (1% normal melting point in PBS), with approximately 1000 cells in 0.5% low melting point agarose in PBS. Slides were lysed at 4 °C in a solution containing 1% Triton X-100, 2.5 M NaCl, 100 mM EDTA, and 10 mM Tris pH 10.0 for two hours. Slides were incubated for 20 min in an alkaline buffer (300 mM NaOH and 1 mM EDTA (pH > 13)) and electrophoresed for 20 min at 20 V and 300 mA at 4 °C in the same buffer. Slides were then neutralized and dried in 70% ethanol before being stained with SYBR Gold (Thermo Fisher Scientific, Waltham, MA, USA) and scored using the tail moment [53].

### 4.6. MTT Assay

Viability was measured with a tetrazolium salt as previously described [52]. The MTT (methyl-thiazol-tetrazolium) assay was used to assess the impact of treatments on cell viability in THP1 cells and PBMCs after 48 h. Cells were incubated with MTT for two hours, lysed in 20% SDS (*w*/*v*), 50% dimethylformamide (*v*/*v*) pH 4.7, and the absorbance was measured at 570 nm.

### 4.7. Statistical Analysis

GraphPad Prism version 6.0 (La Jolla, CA, USA) was used for all calculations. Multiple comparisons were controlled for using the Sidak-Bonferroni method. All experiments were performed at a minimum of 3 independent repeats. Differences with *p*-values less than 0.05 were considered significant.

## 5. Conclusions

Overall this study suggests that doses of Se compounds that achieve plasma Se concentrations in the range of 2.5–5 µM might achieve the optimal balance between enhancing efficacy and reducing the toxicity of radiation. It is possible that higher doses of Se might safely be used with some chemotherapy drugs. It is noteworthy that dosing to achieve plasma Se levels determined by this in vitro study would not apply to seleno-l-methionine, as it is non-specifically incorporated into the general protein pool, especially albumin, which gives disproportionately high plasma Se levels compared to dosing with equivalent elemental Se doses of sodium selenite or Se-methylselenocysteine [54,55].

The potential of Se to improve the efficacy and reduce toxicities of cancer treatments is important and deserves careful examination in clinical trials. However, when designing these trials, we need to be cognizant of the genotoxicity dose-dependence of the Se compounds to be used, with the potential for increased serious late toxicities of cancer treatments such as secondary malignancies, and evaluate this in our trials. Importantly, this study has demonstrated several laboratory methods that can be incorporated into clinical trials to enable investigators to evaluate the pharmacokinetic-pharmacodynamic relationship of the Se compounds being used in cancer patients. This will assist us in rationally determining the optimal dose and form of Se for use in combination with various cancer treatments in clinical trials; such trials are already underway [4,28].

## Abbreviations

AraC	Cytosine arabinoside
Dox	Doxorubicin
GSH	Glutathione
MSA	Methylseleninic acid
PBMC	Peripheral blood mononuclear cell
